# Variable Temporal Length Training for Action Recognition CNNs

**DOI:** 10.3390/s24113403

**Published:** 2024-05-25

**Authors:** Tan-Kun Li, Kwok-Leung Chan, Tardi Tjahjadi

**Affiliations:** 1 Department of Electrical Engineering, City University of Hong Kong, Hong Kong, China; tankunli2-c@my.cityu.edu.hk; 2 School of Engineering, University of Warwick, Gibbet Hill Road, Coventry CV4 7AL, UK; t.tjahjadi@warwick.ac.uk

**Keywords:** deep learning, video classification, action recognition, representation learning

## Abstract

Most current deep learning models are suboptimal in terms of the flexibility of their input shape. Usually, computer vision models only work on one fixed shape used during training, otherwise their performance degrades significantly. For video-related tasks, the length of each video (i.e., number of video frames) can vary widely; therefore, sampling of video frames is employed to ensure that every video has the same temporal length. This training method brings about drawbacks in both the training and testing phases. For instance, a universal temporal length can damage the features in longer videos, preventing the model from flexibly adapting to variable lengths for the purposes of on-demand inference. To address this, we propose a simple yet effective training paradigm for 3D convolutional neural networks (3D-CNN) which enables them to process videos with inputs having variable temporal length, i.e., variable length training (VLT). Compared with the standard video training paradigm, our method introduces three extra operations during training: sampling twice, temporal packing, and subvideo-independent 3D convolution. These operations are efficient and can be integrated into any 3D-CNN. In addition, we introduce a consistency loss to regularize the representation space. After training, the model can successfully process video with varying temporal length without any modification in the inference phase. Our experiments on various popular action recognition datasets demonstrate the superior performance of the proposed method compared to conventional training paradigm and other state-of-the-art training paradigms.

## 1. Introduction

Compared with traditional neural networks, one of the advantages of convolutional neural networks (CNNs) is that they can process inputs of arbitrary size without altering the model structure. However, such an advantage is not fully utilized, as most current models can only process data with one fixed shape that is used during training. Model performance is significantly degraded if the input shape is varied during the testing phase. Therefore, preprocessing of input data is crucial in maximizing performance. For computer vision tasks, resizing is necessary before the images are fed to the network in order to ensure that every sample has the exact same size. Usually, the images are resized to 224×224. As the shapes of different videos and images are naturally different, current preprocessing is suboptimal. Similar to the human visual system, an ideal model should be able to process data of arbitrary size without requiring any resizing in the testing phase.

**Figure 1:**
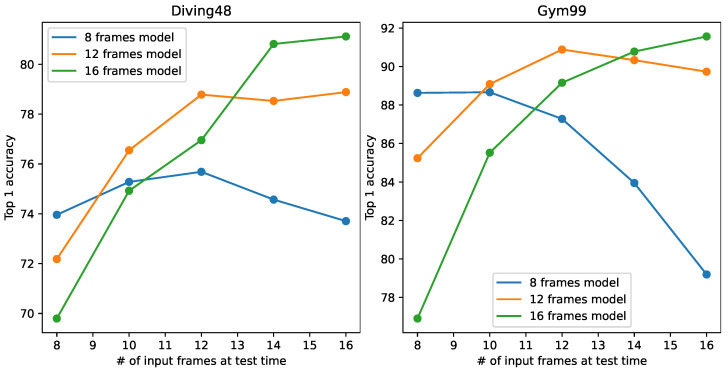
Model performances with different numbers of input frames during testing on Diving48 [1] (**left**) and Finegym99 [2] (**right**). ‘# of input frames’ represents ‘number of input frames’.

In the field of video classification, it is important to unify the temporal length of each sample. Different videos have different lengths. To unify the temporal length, downsampling of video frames is used as an essential preprocessing step. A standard video CNN development pipeline involves the following steps: (1) define the number of frames on which the model is to operate; (2) train the CNN using this number of frames; and (3) test the CNN using this number of frames. Here, the number of frames is fixed and is regarded as a hyperparameter. If the number of sampled frames at test time deviates from that during training, a large degradation in performance will occur. This phenomenon is depicted in Figure 1. We trained our 3D-CNN (model details are discussed in Section 3.1) on Diving48 [1] and FineGym99 [2] using the conventional training paradigm with 8, 12, and 16 input frames. We denote these models trained with different input frame settings as the eight-frame model, twelve-frame model, and sixteen-frame model. After training, the models were tested with different numbers of input frames. Figure 1 shows that the models tended to perform at their best when the testing input frames was set consistently with that used during training. If the train/test settings are different, there can be a huge performance drop. For example, when models trained with sixteen-frame input are used to process samples with eight frames, their performance is much worse than eight-frame models. This suggests that models produced by conventional training only fit the single fixed temporal length used during their training. The more the size deviates from that used during training, the larger the resulting degradation.

The computation cost of video models grows proportionally as the number of input frames increases. Sometimes, the computational demands need to be met by varying the number of frames. The existing training paradigm is unable to yield models with such flexibility, as varying the temporal length degrades the performance. Common practice when developing video models for different temporal lengths is to conduct multiple training using different numbers of frames to produce multiple models, allowing model performance to be evaluated at different frame settings; however, this is both time- and memory-consuming. Moreover, videos naturally have different temporal lengths; if each video is fitted to the same length by downsampling prior to modeling, longer videos may lose a great deal of information, resulting in the model having insufficient cues to make a correct prediction. Moreover, when a video is downsampled to a different number of frames, the feature pattern is changed. Requiring the input frames to be of one fixed length during training limits model performance due to a lack of richness on the part of the feature pattern.

In this work, we propose an effective new training paradigm for producing 3D-CNNs that can flexibly fit video of varying temporal length during testing. One approach is to train one model at each temporal length, save all of them, and pick the designated model that is required at test time, which is the adopted practice in most works (e.g., [3,4,5]). As discussed before, this practice is clearly not ideal. Intuitively, in order for a 3D-CNN to perform at different temporal lengths, it is only necessary to train the model with varying training samples. However, due to the need for parallel training and normalization shifting, this is not a trivial task.

Graphics processing units (GPUs) have been heavily used to accelerate the training of deep learning models. A GPU expects batches of data with the same shape at each training iteration on which to perform parallel computing. If each sample has a different temporal length, these samples cannot be stacked together for use by the GPU. Therefore, the straightforward solution of vanilla variable input length training (VVLT), which randomly alters the temporal length for each sample during training, cannot be applied to training on GPUs. For example, it can be ensured that the temporal lengths are consistent within the same training iteration or training epoch while allowing them to vary at different iterations or epochs; we denote these two training methods as VVLT-iter and VVLT-epoch (further details are provided in Section 4.4). However, it is not easy to vary the temporal length efficiently at the iteration level with a multi-worker data loader in an existing deep learning library (e.g., Pytorch [6]). Moreover, the normalization shifting problem occurs as the temporal length changes, which means that there is a discrepancy in batch normalization (BN) statistics at different frame settings. This phenomenon is discussed in detail in [7].

Varying the temporal length at the iteration/epoch level makes training unstable, as the BN statistic is different at different iteration/epoch numbers. In our experiments, we show that these simple solutions do not work as expected. Apart from these straightforward methods, there have been a few attempts to develop a better flexible training paradigms [7,8]. While these methods perform better than traditional training methods, there is room for further improvement in terms of algorithmic complexity and prediction accuracy.

**Figure 2:**
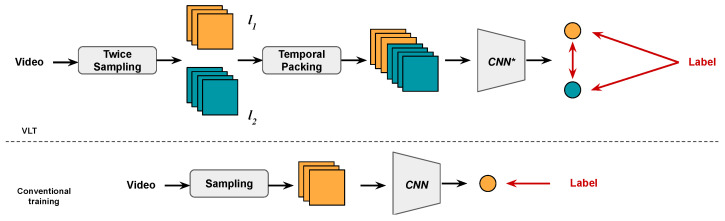
Comparison between the proposed method (**upper figure**) and conventional training methods (**lower figure**). The black arrows represent the tensor flows and the red arrows represent loss computation, while CNN* represents a modified CNN.

In this paper, we propose a simple yet effective training paradigm that grants models the ability to process inputs with varying lengths during testing. An overall illustration of the proposed method is shown in Figure 2. Compared with conventional training, there are four differences in our method. First, instead of sampling just once for each training sample, we sample twice, resulting in two downsampled videos, which we denoted as separate subvideos. We denote the length of these two subvideos as $l_{1}$ and $l_{2}$. During training, we allow $l_{1}$ and $l_{2}$ to be different. Second, after sampling twice, the temporal packing procedure concatenates the two subvideos in the temporal dimension to form one training sample. The total temporal length of this training sample is $L=l_{1}+l_{2}$. We fix the total length *L* during training. Third, the training sample is fed to a modified 3D-CNN with subvideo-independent 3D convolution layers. Such modifications ensure that the data from different subvideos do not interfere with each each other, which is the key design of our training method. Note that we only need to modify the 3D convolution layers during training; no modification is needed during testing. Fourth, in addition to the common classification loss computed between outputs and labels, we introduce a new consistency loss between the different subvideos to reinforce the prediction consistency with inputs of varying lengths. Compared with conventional training, the proposed VLT has several advantages: (1) during training, $l_{1}$ and $l_{2}$ (the temporal lengths of the subvideos) are randomly determined and can be different at each training iteration, allowing the models to adapt to videos of varying length; (2) as the models can now learn from richer feature patterns, they are able to achieve higher accuracy than in conventional training; (3) because the total length of each sample *L* is fixed, the BN layers can be updated stably and the normalization shifting problem is resolved, while fixing the value of *L* means that each sample has the same shape, and can be stacked together and trained on GPU; and (4) in addition to the VLT structure, our new consistency loss further refines the representations. Compared with other flexible training methods, our method is cheaper in both implementation and training, and yields good results when adapting to videos with varying lengths. The contributions of this paper are as follows:

- We propose a new 3D-CNN training paradigm for video classification tasks. Multiple techniques (sampling twice, temporal packing, subvideo-independent convolution) are introduced to assist the model in learning to process videos of different temporal lengths while improving classification accuracy.
- To further increase performance, we introduce a consistency loss that reinforces the prediction consistency between subvideos of different lengths sampled from the same video.
- Unlike previous works that have attempted to enable deep models to process input of varying shapes by improving the capacity of the model (e.g., adaptive layers [7,9]), our method does not need any modifications at test time. This suggests that the commonly used training paradigms are suboptimal and limit the potential of deep models.
- Extensive experimental results show the superiority of the proposed method over the conventional training paradigms as well as alternatives, with better performance, cheaper training cost, and simpler implementation.

## 2. Related Work

### 2.1. Video Recognition

As more and more video data are created and stored, understanding these data is becoming increasingly important. Recently years have witnessed enormous development of video models. In the past, clumsy CNN models with heavy 3D convolution layers, such as C3D [10] and I3D [11], were widely studied. These models require enormous amounts of computation and are expensive to train, making them difficult to deploy in real life. To address this, lightweight 2D-CNN models equipped with cheap temporal interactive operations have been proposed, e.g., TSM [3], TAM [12], TDN [4], GC [13] and X3D [14]. These models are based on ResNet [15] and EfficientNet [16], and the computation cost of the temporal modelling layers is often negligible. Due to their simplicity of implementation and inexpensive computation, these models have become the most popular in practice. These methods can even be designed for efficient deployment on edge devices [3,14]. With the advancement of Vision Transformer [17,18], more and more video models based on the transformer structure are being developed, e.g., Uniformer [5], FocalNet [19], and Video-Swin [20]. While these transformer-based models are slightly better than CNNs in terms of accuracy, they require more computation and execution time for the self-attention layers.

### 2.2. Flexible Input Shape Inference

Unlike the human visual system, most deep models can only perform inference on a fixed input shape. Images, videos, texts, etc., are all data types that are naturally of different shapes. A deep model that can only perform inference on a fixed shape is clearly suboptimal. Recently, more and more attention has been paid to developing non-static models that can adapt to any input shape during inference. For example, continuous kernel convolution [9] substitutes the conventional convolution, allowing the kernel weights to be adapted according to the sequence length. In language modelling, example packing [21] has been proposed as a way to efficiently tackle the limitation of fixed input length training.

In computer vision, attempts are increasingly focused on enabling models to process varying input shapes. FlexVit [22] enables Vision Transformer (ViT) to flexibly change the patch size during inference, resulting in more adaptive computation costs. NaViT [23] takes inspiration from language model and trains the image model with example packing, which accelerates the training process while enabling the model to perform at varying image sizes.

The above-mentioned methods are specifically designed for transformer-based models intended for image classification tasks. For understanding videos, MutualNet [8] has been proposed for training a single model that can run under diverse resource constraints by exploiting multi-stream training and knowledge distillation. Note that this mutual learning scheme allows networks to take in varying input shapes as well as to alter the size of the network itself. FFN [7] can be regarded as an improved version of MutualNet, with partially shared networks used to perform evaluation on different frame settings. Thus, while FFN has more parameters than MutualNet, it enjoys significantly improved performance. Unlike these two works, our method is much simpler in implementation, has lower training costs, and does not require any extra parameters. More detailed comparisons are presented in Section 4.

## 3. Method

We aim to develop a better training scheme that can be applied to any CNN for video classification tasks. In this section, we first introduce the 3D-CNN used in this paper, followed by the proposed method for variable length training (VLT). Pytorch v2.3 [6] is used in this paper.

### 3.1. Temporal Partial Convolution (TP Conv)

**Figure 3:**
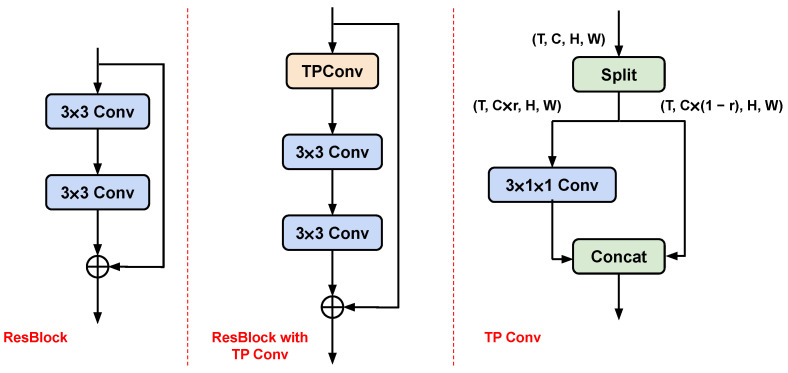
Illustration of residual blocks in ResNet18 [15] (**left**), the residual block proposed in this paper (**middle**), and the details of temporal partial convolution (TP Conv) (**right**). The text in brackets describes the shape of tensors.

We start by introducing the 3D-CNN used in this work. We want to show that our training method can be easily applied to 3D convolution. Regular 3D convolution incurs a tremendous amount of computation. On the other hand, popular light weight video classification CNNs such as MVF [24] and ActionNet [25] include many depth-wise temporal convolutions, which execute more slowly than regular convolutions (especially on GPU), though with lower FLOPs (floating point operations) [26]. To achieve fast training and light weight at the same time, we take inspiration from FasterNet [26], extending the idea of partial channel convolution to temporal modelling and building temporal partial convolution (TP Conv). Unlike regular convolution, partial convolution only conducts a convolution operation on part of the channel, leaving the rest untouched. In this way, the convolution layers can achieve lower latency while still producing decent performance. The TP Conv in this work is shown in Figure 3. A TP Conv is inserted in the standard residual block. Given a tensor of shape T×C×H×W (i.e., temporal length, channel size, height, and width), TP Conv first splits the tensor in the channel dimension, resulting in two tensors of shape T×C∗r×H×W and T×C∗(1−r)×H×W. The hyperparameter *r* denotes the number of channels used for convolution. As architecture design is not the focus of this paper, we set r=0.25, as in the original paper. TP Conv is inserted in each of the residual blocks in ResNet18. We demonstrate comparative results between TP Conv and other popular 3D network in Table 1, showing that TP Conv can achieve good performance without too much execution time.

Note that TP Conv is not the main contribution of this work; rather, it is a workaround to achieve faster training on GPU without losing accuracy. Our method can be easily extended to other 3D-CNN structures. The performance of our method using other CNN structures is shown in the experiments section.

### 3.2. Variable Length Training (VLT)

**Figure 4:**
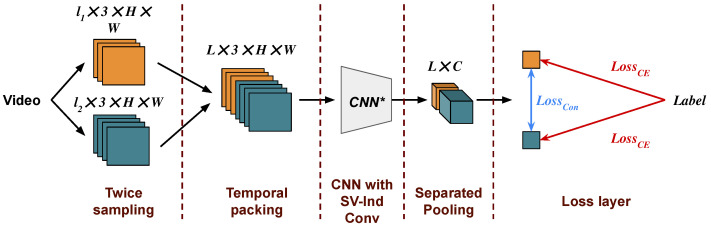
The proposed VLT. The text at the top describes the shape of the tensors. SV-Ind Conv denotes subvideo-independent convolution.

The conventional training paradigm can only yield models that function for a single number of frames, which introduces various inconveniences. In this section, we propose a new training paradigm for video understanding, VLT, which enables the model to process videos with a wide range of number of frames. A more detailed illustration of our method is shown in Figure 4. Our method can be divided into five parts: sampling twice, temporal packing, an SV-Ind CNN, a separated pooling layer, and a loss layer.

#### 3.2.1. Sampling Twice

In the conventional training paradigm, only one subvideo with a fixed length is sampled from the full video, which results in the model only operating at a fixed video length. In the proposed method, we first define the range of the number of frames that we want the model to operate on as $S_{n–m}=\left[n,n+1,n+2,\ldots ,m\right]$; for instance, n=4 and m=16 means that after training the model should function with any frame setting between 4 and 16. The total temporal length of each training sample is then defined as L=n+m. For each sample, we sample two subvideos from the full video instead of one at each training iteration; the temporal lengths of these two subvideos are denoted as $l_{1}$ and $l_{2}$. Specifically, $l_{1}$ is first randomly drawn from $S_{n–m}$, then $l_{2}$ is computed by $l_{2}=L-l_{1}$. The shapes of the two subvideos are $l_{1}\times 3\times H\times W$ and $l_{2}\times 3\times H\times W$, respectively, with a channel size of 3 for RGB frames.

#### 3.2.2. Temporal Packing

After the sampling layer, the two subvideos are concatenated in the temporal dimension, which we refer to as temporal packing. After packing, all the samples during training share the exact same shape L×3×H×W, which enables the model to be trained via GPU. Intuitively, to enable the model to deal with inputs of variable length, training the model with such variable length inputs should solve the problem, just as in VVLT-iter or VVLT-epoch. However, we found that models trained with these methods remained unable to process variable length video (see the results in Section 4.4). This is because when varying the input length at the iteration level or epoch level, the BN statistics are different at the iteration level or epoch level, which makes the whole training unstable. FFN was proposed in [7] to address this issue using unique BN layers, sharing only part of the convolution layers when provided with different frame numbers. Here, we instead solve the issue by unifying the total temporal length *L* for each training sample without altering or adding any new parameters to the networks. In this way, the BN statistics become stable at every training iteration and the discrepancy is eliminated.

#### 3.2.3. Modification of Convolutions during Training

**Figure 5:**
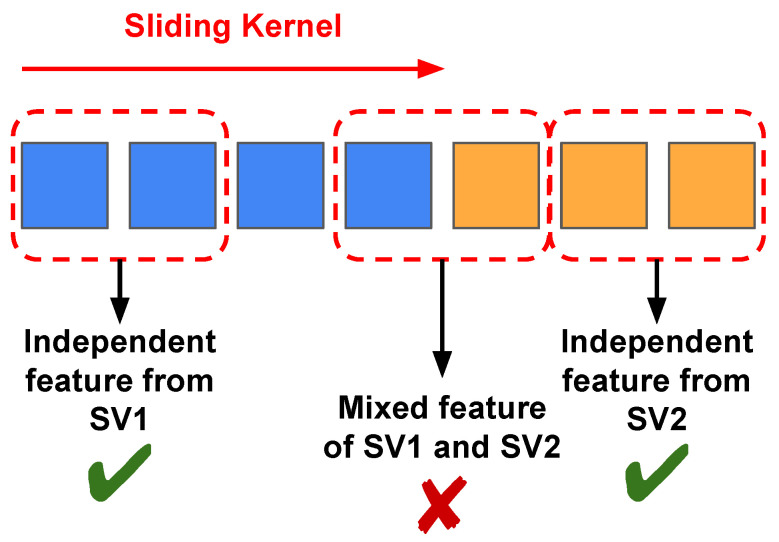
Regular temporal convolution. The blue and orange rectangles are two subvideos concatenated in the temporal dimension. The red dotted rectangles represent three positions of the temporal convolution kernel that slides from left to right. The convolution kernel size is 2 in this case, while SV1 and SV2 are short for subvideos 1 and 2. ✔ represents the output feature at that position is correct and × represents incorrect.

After temporal packing, each training sample contains two subvideos of different lengths. We want the model to process these two independently despite them being within the same training samples. This step is very important; if regular convolution layers are used during training, the model will treat the two subvideos as related frames in the same video and learn the incorrect representation. This phenomenon is illustrated in Figure 5. The figure shows a training sample, containing two subvideos, after the temporal packing procedure. When a temporal convolution with kernel size 2 is used for temporal modelling, the convolution layer models the last frame of subvideo 1 and the first frame of subvideo 2 jointly at the junction. Preventing the features from interfering with each other is an easy task in transformer-based architecture, as masks can simply be applied to the attention matrix in the self-attention layers [21]. Here, we introduce subvideo-independent convolution (SV-Ind Conv) for 3D-CNN structures. There are two easy ways to implement SV-Ind Conv. A standard 3D convolution with kernel size 3 is provided by

(1) $$y_{t}=w_{1}x_{t-1}+w_{2}x_{t}+w_{3}x_{t+1},$$

where $y_{t}$ and $x_{t}$ respectively denote the output and input features at frame *t* and $\left[w_{1},w_{2},w_{3}\right]$ is the kernel weights of convolution. When using this regular convolution in our method, the features interfere with each other incorrectly when $x_{t-1}$ or $x_{t+1}$ is from a different subvideo than $x_{t}$. To address this, the convolution can be modified as follows:

(2) $$y_{t}=\beta_{t-1}*w_{1}x_{t-1}+w_{2}x_{t}+\beta_{t+1}*w_{3}x_{t+1}$$

where two binary weights $\beta_{t-1},\beta_{t+1}\in \left[0,1\right]$ are added. We set $\beta_{t-1}$ or $\beta_{t+1}$ equal to 1 if $x_{t-1}$ or $x_{t+1}$ is from the same subvideo as $x_{t}$; otherwise, $\beta_{t-1}$ or $\beta_{t+1}$ is set to 0. This implementation requires rewriting some code in the deep learning libraries for the built-in convolution functions, as the basic computation is changed; however, this can be accomplished easily.

**Figure 6:**
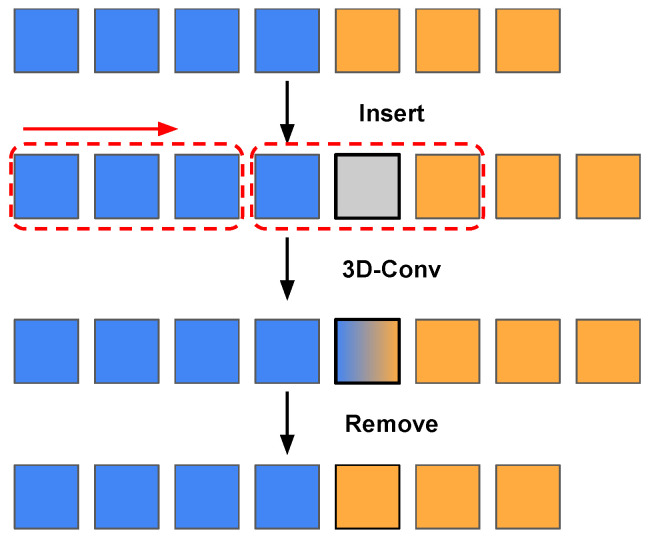
Insert-and-Remove operation for SV-Ind Conv. The blue and orange rectangles are two subvideos concatenated in the temporal dimension, the grey rectangle is the empty frame inserted in between, and the rectangle with gradient colour is the output of 3D convolution where the features from the two subvideos are mixed together. The red rectangles are two examples of sliding convolutional kernels with kernel size 3. The zero padding on both sides is ignored in this figure.

Here, we provide a second implementation that does not need any modification for the convolution functions, which we call Insert-and-Remove. An illustration of this process is provided in Figure 6. Given a training tensor of size L×C×H×W, we first insert ⌊K/2⌋ empty frames (with all elements equal to 0) between the two subvideos, where *K* is the kernel size and ⌊·⌋ is the floor function. After insertion, we have a tensor of size (L+⌊K/2⌋)×C×H×W, which is then processed by a standard 3D convolution layer. After convolution, the inserted empty frames become the corrupted feature where the two subvideos are jointly modelled; these corrupted frames are then removed. In this way, the two subvideos are modelled independently despite being in the same training sample. This implementation is much easier than the first; although it requires slightly more computation cost on the empty frames, this can be considered negligible.

The above two implementations are equivalent to each other. In this work, we use the second implementation due to its simplicity. Note that the SV-Ind Conv is only needed during training when multiple subvideos are combined together. In the evaluation phase, where there is only one subvideo in each sample, this modification is unnecessary.

#### 3.2.4. Separated Pooling

In all the video classification models, there is a global pooling layer that squeezes the feature in spatial and temporal dimensions and produces a one-dimensional representation vector. This global pooling is usually undertaken by simply averaging all the elements. In our work, this layer needs modification during training, as there are two subvideos within one sample. We simply split the two subvideos before the global pooling layer to keep these two representations separate.

#### 3.2.5. Loss Layer

**Figure 7:**
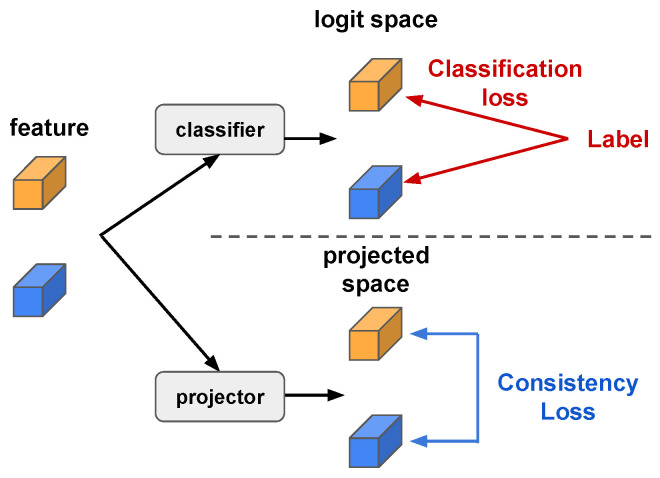
Detailed view of the proposed loss layer. The cuboids of different colours on the left represent the one-dimensional vector of two subvideos. Both the classifier and the projector are simple fully connected layers.

In the conventional training paradigm, there is only one representation for each training sample; thus, only one classification loss is needed. In our method, we have two representations for each training sample; thus, we accordingly modify the loss layer to improve the performance. A more detailed loss layer is illustrated in Figure 7. There are two loss terms in our method. The first loss is the usual classification loss, which computes the cross entropy between the output and label. The classifier is a fully connected layer that produces a logit vector for final classification. The classification loss can be provided by

(3) $$L_{cls}=\frac{1}{2}\left(L_{CE}\left(x_{1},y\right)+L_{CE}\left(x_{2},y\right)\right),$$

where $x_{1}$ and $x_{2}$ are logit vectors of the two subvideos, *y* is the label, and $L_{CE}$ is the standard cross-entropy loss for classification task. Note that we ignore the softmax operation here for simplicity.

The second loss, i.e., the consistency loss, is designed to ensure consistency of representation between subvideos of different temporal lengths as well as between different samples that are from the same class within one batch. Given the feature vector of two subvideos, another fully connected layer projects the vectors to another space. This projector takes in a feature vector of size *C* and outputs a projected feature of the same size. Here, we consider this loss from a batchwise perspective. We denote $Z_{1}\in R^{B\times C}$ and $Z_{2}\in R^{B\times C}$ as batches of the projected vector of the first and second subvideos, where *B* is the batch size. We compute the relations between different subvideos within the same batch by

(4) $$R={\bar{Z}}_{1}\cdot {\bar{Z}}_{2}^{T},$$

where $R\in R^{B\times B}$ represents the relations between different subvideos and ${\bar{Z}}_{1}$, ${\bar{Z}}_{2}$ represent the normalized projected feature. To construct the label of this relation, we convert the ground truth to a one-hot vector label and compute the relation label by

(5) $$Y_{R}=\hat{Y}\cdot {\hat{Y}}^{T},$$

where $Y_{R}\in R^{B\times B}$ is the relation label and $\hat{Y}\in R^{B\times N}$ (with *N* as the total number of classes) is a mini-batch of the one-hot label vector. Finally, the consistency loss is written as a symmetric cross entropy loss [27], i.e.,

(6) $$L_{con}=\frac{1}{2}*\left(L_{CE}\left(\frac{R}{\tau },\frac{Y_{R}}{\sum_{j}{Y_{R}}_{\left(i,j\right)}}\right)+L_{CE}\left(\frac{R^{T}}{\tau },\frac{Y_{R}^{T}}{\sum_{j}{Y_{R}^{T}}_{\left(i,j\right)}}\right)\right),$$

where τ is the temperature, which controls the range of the logits in the softmax. The overall loss of the proposed work can be provided by

(7) $$L=L_{cls}+\alpha L_{con},$$

where α is the weight of the consistency loss. In this way, we ensure that the model produces consistent representations when provided with videos of different lengths as well as when provided with different samples that belong to the same class.

## 4. Experiments

### 4.1. Dataset

We first evaluated the effectiveness of the proposed method and compared it with other training paradigms on Diving48 [1] and FineGym [2]. These two datasets are fine-grained motion recognition datasets that focus on human motions. There are about 18,000 videos covering 48 competitive diving classes in Diving48, with 15,000 samples in the training set and 2000 samples in the testing set. There are two different splits in Finegym, i.e., Gym99 and Gym288, involving 99 classes and 288 classes of gymnastic actions, respectively. For Gym99, there are about 20,000 training samples and 8500 testing samples. For Gym288, there are 23,000 training samples and 9600 testing samples. Later, we extended our method to more popular datasets: UCF101 [28], HMDB51 [29], and HAA500 [30]. UCF101 is a classic and widely used dataset, with 13,320 videos from 101 action categories. HMDB51, another popular dataset, is collected from movies and the internet, containing about 7000 videos divided into 51 classes. HAA500 is a relatively new dataset concentrating on human-centric atomic action in 500 classes, with about 590,000 labeled frames. There are multiple training–testing splits in UCF101 and HMDB51. We used the first split for both of these two datasets.

### 4.2. Implementation Details

We followed the common training recipe for video classification models from [31]. Frames were uniformly sampled from each video and re-scaled to 256×256. Each frame was randomly cropped to 224×244 during training, while the centre crop was applied during testing. Random horizontal flipping was used for data augmentation when training. For all models on all datasets, we trained the model for 40 epochs in total using SGD with momentum 0.9 and weight decay 0.0005. The learning rate, initialized as 0.01, was decayed by 0.1 at epochs 20, 30, and 35. The batch size was set to 32.

For the hyperparameter in VLT, we set the range of frame numbers to $S_{n–m}=S_{8–16}=\left[8,9,\ldots ,16\right]$ as the default, i.e., the model was trained to adapt to any frame numbers between 8 and 16. This setting was altered for later experiments to provide more in-depth analysis. The temperature and the weight of consistency loss were set to τ=0.05 and α=0.4 unless otherwise specified.

### 4.3. Comparison with Basic Training Paradigm

In this section, we compare the proposed method with the conventional training paradigm. A common metric used to evaluate model performance is Top1/Top5 accuracy. Usually, models are trained and evaluated at a fixed frame setting. In our work, the frame setting can vary during evaluation and the model needs to be evaluated at different frame settings. This common metric is unable to reveal the capacity to adapt to different number input frames straightforwardly. Thus, we introduce a new metric for evaluating the ability of a model to adapt to varying input length δ. Given a model *M*, its δ is computed by

(8) $$\delta_{M}=\frac{\sum_{j\in F}\left(A\left(M,j\right)-A\left({\hat{M}}_{j},j\right)\right)}{\#F},$$

where A(M,j) is the Top1 accuracy of model *M* on *j* frames input, ${\hat{M}}_{j}$ represents the model trained with *j* frames input with conventional method, *F* is a set of frame numbers used in the evaluation, (e.g., F=[2,4,6,…,16]), and #F denotes the size of set *F*. In Section 1, we show that models tend to be at their best when the frame settings are consistent in training and testing, i.e., the best performance that can be obtained at frame number *j*, which is $A({\hat{M}}_{j},j)$, is produced by model ${\hat{M}}_{j}$. In a nutshell, δ demonstrates the difference between the performance of a model at different frame settings and the best performance that can be obtained at different frame settings. If δ<0, it means that the model is unable to adapt to varying temporal length, while if δ≥0, then the model can adapt to inputs of varying lengths that are within set *F*.

**Figure 8:**
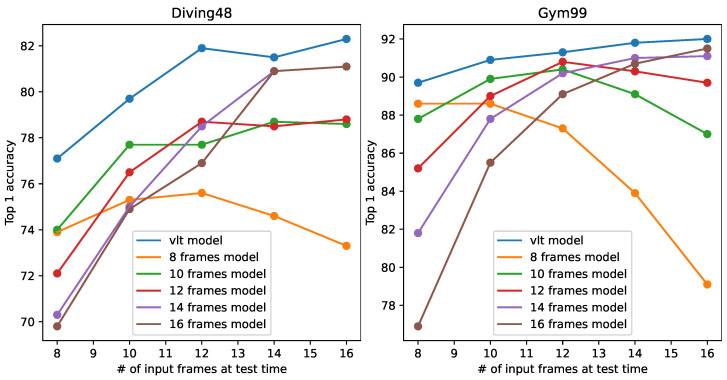
Comparison with the conventional training paradigm on Diving48 [1] (**left**) and Gym99 [2] (**right**). ‘# of input frames’ represents ‘number of input frames’.

We show the comparison between the common training paradigm and the proposed training paradigm in Table 2 and Table 3 and in Figure 8. The tables and figure evaluate the model performance with eight frames and sixteen frames (i.e., F=[8,10,12,14,16]). Looking at the conventionally trained models, in these two tables it can be observed that the models are not able to adapt to different frame settings when performing inference (δ<0). Moreover, δ tends to be higher for models trained with medium lengths (models 10f to 14f) and lower for models trained with small and large lengths (models 8f and 16f). This can be interpreted by the average pattern discrepancy between training and testing input. If the frame setting is close to that used in training, the feature pattern will be similar. Therefore, the more the testing frame number deviates from that used during training, the more degradation will be seen. Obviously, a model trained with medium length will experience less pattern discrepancy compared to a model trained with smaller or larger lengths. Looking at the ensemble model, where we saved all the conventionally trained models and picked the most suitable when given different frame numbers at testing, the model is able adapt to different frame settings, though at the cost of 5× the training time and 5× the parameter size. Ensemble methods are the most widely used evaluation approach for different frame numbers when developing new video classification models (e.g., [4,5,13,14]); however, due to the high degree of inconvenience, most works have only conducted evaluations at eight and sixteen frames.

For the proposed VLT, δ is greater than zero on both datasets, which means that the proposed training paradigm can operate with any frame numbers between 8 and 16 while further improving the performance compared to the commonly used ensemble model. There are two reasons for this. First, videos of varying lengths have different feature patterns; for conventional training, the model is only trained on one fixed temporal length with a limited feature pattern. With the proposed VLT, the model is trained on a much richer pattern and can learn a better representation. Second, in addition to the classification loss, we introduce an extra consistency loss, which ensures that the model produces stable and consistent predictions when provided with inputs of different temporal lengths and with different samples within the same class. This loss term further improves the representation quality. More details are provided by the ablation study on consistency loss.

For a more straightforward demonstration, we depict the accuracy curve for varying frame numbers in Figure 8. The figure shows that the models trained with the conventional method degrade significantly when the frame settings in the testing phase deviate from those used during training. For the proposed method, the model trained with VLT always outperforms others at any given frame number. These results demonstrate the superiority of VLT over the conventional training paradigm.

### 4.4. Comparison with Flexible Training Paradigm

In this section, we compare our method with four types of flexible training paradigm which aim to enable models to process videos of varying lengths. First, ensemble methods are a common practise for processing varying length videos, where training uses multiple videos at different frame numbers and the most suitable one is then chosen for inference. Second, VVLT-iter and VVLT-epoch have already been introduced in Section 1. For VVLT-iter, a temporal length from 8 to 16 is randomly drawn for each iteration. Similarly, the temporal length of VVLT-epoch is randomly drawn from between 8 and 16 for each epoch; that is, for VVLT-iter/epoch, the temporal length is the same within the same iteration/epoch but can be different at different iterations/epochs. Third, MutualNet [8] was the first adaptive 3D-CNN for video classification tasks. MutualNet uses a mutual learning scheme to train a single model under different configurations in a multi-stream manner. It utilises the knowledge distillation loss to enforce consistency. Note that this work mainly focuses on image classification, not video classification. To ensure a fair comparison, we only varied the temporal resolution, not the image spatial size or network size. The temporal resolution factor used in this paper for MutualNet was the same as in the original paper. Fourth, FFN can be regarded as an improved version of MutualNet developed specifically for video classification. We strictly followed the original work to produce our results. All the results were obtained using the same model and same framework.

**Figure 9:**
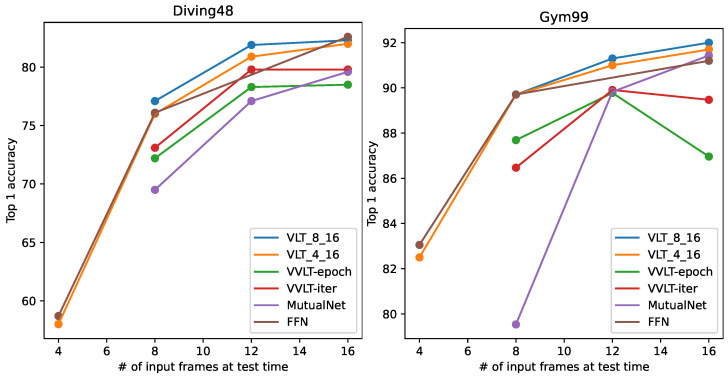
Comparison with other flexible training paradigms and state-of-art methods on Diving48 (**left**) and Gym99 (**right**).

We show the results on Diving48 and Gym99 in Table 4 and Table 5. In these two tables, it can be seen that VVLT methods do not work well when adapting to different frame sizes (δ<0). This is due to the normalization shifting phenomenon. During training, when the input length is varied at the iteration/epoch level, the BN statistics vary at the iteration/epoch level as well, which introduces instability during training. It can be seen that MutualNet scores poorly on these two datasets. There are two reasons for this. First, this method is mainly designed for image tasks, and generalizes poorly to video tasks. Second, in the original work, only one video dataset (Kinetics [11]) was used for evaluation, making for an appearance-dominant dataset. Both Diving48 and Gym99 are motion-dominant datasets that stress temporal information. For appearance-dominant datasets, models make predictions by mainly focusing on 2D features; thus, varying temporal lengths, which significantly change 3D features, do not affect performance very much. For motion-dominant datasets, where the model needs to find cues from 3D features, varying lengths make a huge difference in the features. For deep models, processing videos of varying length in a motion-dominant dataset is much more difficult than when using an appearance-dominant dataset. A method that works on appearance-dominant datasets is not guaranteed to work on motion-dominant datasets. This phenomenon is further discussed in [7]. Unlike the above methods, FFN works adequately with δ=1.4 on Diving48 and δ=0.2 on Gym99. Compared to the proposed method, FFN scores better at a low frame number (4 frames) on both datasets and at a high frame number (16 frames) on Diving48. This is because in the original paper [7] FFN was designed to learn only from videos with 4, 8, and 16 frames; thus, FFN can only process these three frame settings. On the other hand, our method trains the model (VLT-$S_{4–16}$) with a wider range (all possible frame numbers between 4 and 16). Therefore, FFN sometimes performs better when the frame number equals 4, 8, or 16, but performs worse than our method otherwise. In addition, our method has a higher δ than FFN, suggesting that it performs better than FFN and is capable of processing a wider range of frame settings in general. For the proposed VLT, our method succeeds in adapting to a wide range of frame numbers. We trained the model with VLT under two different ranges ($S_{8–16}$ and $S_{4–16}$). All the results obtained with VLT can achieve δ>0. The accuracy curves at different frame settings are plotted in Figure 9. The figure shows that both VLT and FFN largely outperform the other training paradigms, while our method is slightly better than FFN.

To further demonstrate the advantages of our method, we conducted further comparisons of different methods in terms of training complexity (Table 6) and implementation complexity (Table 7). Note that we used the same model with different training methods in these two tables; thus, these methods all have the same inference complexity and different training complexity. Looking at the training complexity, the ensemble method is obviously the most inconvenient, as requires training one model for every frame number. For the other flexible training paradigms, the training cost is significantly reduced. The training FLOPs records the number of floating point operations needed for training each sample. In this paper, the total temporal length *L* is introduced to assist the learning; thus, while our method surely has more training cost than a single model trained using a conventional method (e.g., 16f-model in Table 6), it has lower computation cost compared to the other flexible training methods. For the number of parameters, the ensemble method contains multiple models; therefore, it comprises significantly more parameters than the other methods. FFN specializes the BN layers and some of the convolution layers to help the model adapt to different frame settings; thus, it also requires extra parameters. For training time and GPU memory, both MutualNet and VLT-$S_{4–16}$ achieve the best results among the flexible training methods; however, it should be noted that MutualNet fails to process videos with varying lengths on both Diving48 and Gym99. Compared with FFN, our method can train the model faster while occupying less GPU memory.

With regard to the implementation complexity (Table 7), the ensemble method again performs the worst, as the entire model parameters need to be changed when provided with different frame settings during both training and testing. FFN only modifies the BN layers and some convolution layers when provided with different video lengths, not the entire model, resulting in partial sharing. Therefore, FFN needs some modification to the model when provided with different frame settings during training and testing. For VLT, there modification is only needed during training (SV-Ind Conv), and such modification can be very easily done without invoking any extra computation cost. During the testing phase, our method needs no modification. In general, compared to the other flexible training methods, VLT is more efficient and cheaper in terms of both training and implementation complexity.

### 4.5. Results on Other Datasets

**Figure 10:**
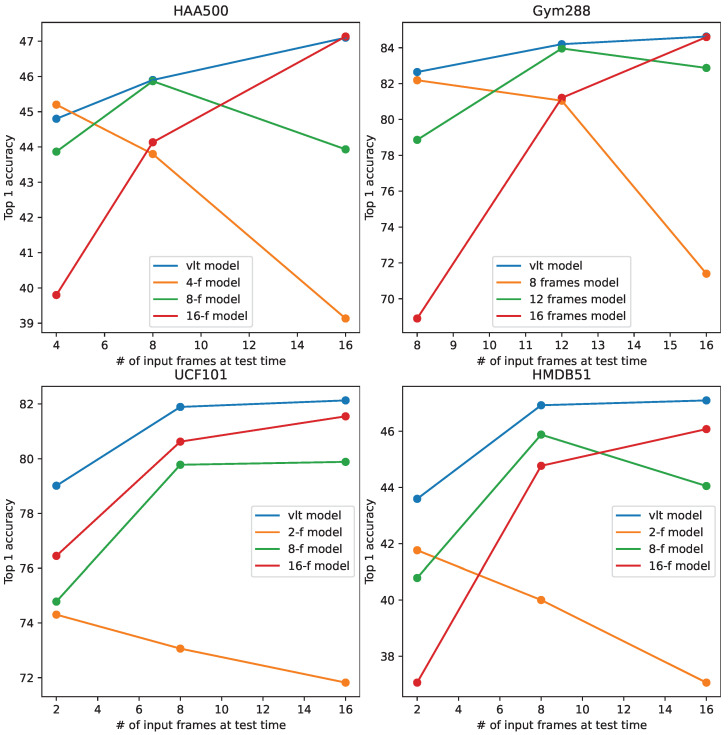
Results compared with the conventional training paradigm on HAA500 [30], Gym288 [2], UCF101 [28], and HMDB51 [29].

In this section, we verify the proposed method on other popular datasets. Figure 10 shows that the observations in Section 1 still hold on four additional datasets (HAA500 [30], Gym288 [2], UCF101 [28], and HMDB51 [29]), that is, the models produced by the conventional training paradigm tend to perform at their best when the frame numbers are consistent between training and testing, while their performance degrades otherwise. Compared to conventional training, our method generally performs better at any frame number. Note that for the datasets used in this paper, Diving48, Finegym, and HAA500 are motion-dominant datasets, while UCF101 and HMDB51 are appearance-dominant datasets. These results show that the proposed method generalizes well among different types of video datasets.

### 4.6. Ablation Study of Consistency Loss

In this section, we evaluate the effectiveness of our proposed consistency loss. We conducted experiments with different weights α for the consistency loss, with the results shown in Table 8 and Table 9. When α=0, which means that only the classification loss is used during training, our method still outperforms the ensemble model and is able to generalize to different frame numbers. When increasing α, the overall performance is improved, which is more obvious on Diving48, where α=0.4, than on Diving48 (where δ=2) or Gym99 (where δ=0.8). Another observation is that the consistency loss helps the model to better adapt to very low frame numbers (i.e., 8). It is common for input with low frames to contain insufficient cues for the model to make the correct prediction. With the help of the proposed consistency loss, which enforces the consistency between videos of different lengths, the model learns to recognize low frame inputs better. These results demonstrate the effectiveness of the proposed consistency loss.

### 4.7. Effectiveness of SV-Ind Conv

**Figure 11:**
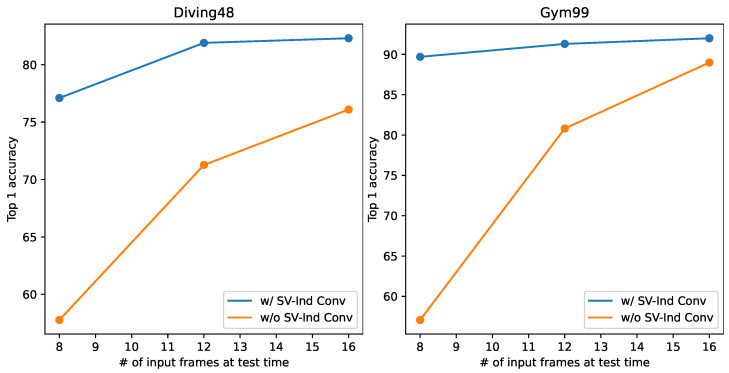
Performance of VLT during training with and without SV-Ind Conv.

To demonstrate the importance of SV-Ind Conv during training with VLT, we provide the curves of training with and without SV-Ind in Figure 11. The figure shows that performance is significantly degraded when training without SV-Ind Conv, especially at low frame numbers. This is because if SV-Ind Conv is not applied when training, the features of the two subvideos interfere with each other and the model learns incorrect representation. Therefore, it is important to use SV-Ind Conv, which is very simple to implement and does not involve very much added computation cost.

### 4.8. Using Different Models

**Figure 12:**
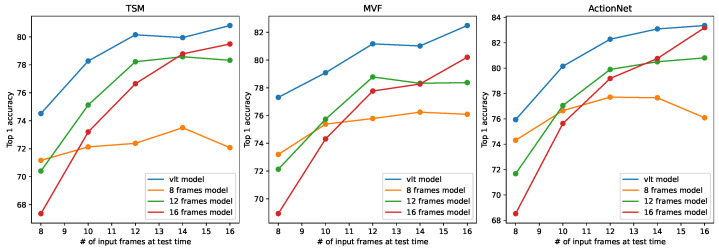
Results of other popular action recognition models trained with VLT on Diving48.

**Figure 13:**
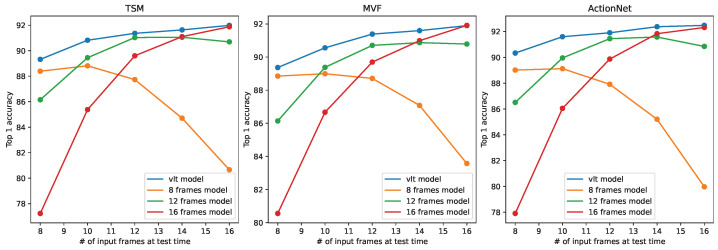
Results of other popular action recognition models trained with VLT on Gym99.

To evaluate the generalization ability of the proposed training method on different CNN structures, we conducted more training on other state-of-art action recognition models, such as TSM [3], MVF [24], and ActionNet [25]. As the temporal modelling modules of these models are simple variants of 3D convolutions, our method can be simply applied to them. The results are shown in Figure 12 and Figure 13. All these models were trained as in the original papers, i.e., the conventional training paradigm. The two figures clearly show that the proposed training method enables the models to operate at a wider range of temporal length while improving their prediction accuracy. These results illustrate the generalization ability of the proposed method.

## 5. Experiments on Large-Scale Dataset

In this section, we present the results of experiments on the Something-Something V1 [32] dataset using two popular models, namely, TSM [3] and MVF [24]. The results are shown in Table 10. Compared to the conventional training method, the proposed VLT performs better in terms of Top1 accuracy. Furthermore, to obtain models operating at different numbers of frames, multiple training procedures need to be conducted for conventional training (i.e., the eight-frame model and sixteen-frame models need to be trained separately). For VLT, however, the model only needs to be trained once, and can perform at different numbers of frame inputs.

## 6. Limitations

The experimental results presented in this paper show that the proposed VLT enables 3D-CNNs to operate on videos in a much wider range of temporal lengths compared with the conventional training, where the trained model can only function at one fixed temporal length. However, the limitations of this approach can be seen in Table 4 and Table 5, i.e., increasing the range of set $S_{n–m}$ leads to a drop in δ. When $S_{8–16}$ is changed to a wider range $S_{4–16}$, δ drops from 2 to 1.5 on Diving48 and from 0.8 to 0.5 on Gym99. This suggests that if set $S_{n–m}$ is extremely large, VLT may not work very well. We believe that this is due to the huge discrepancy between the very low frame number features and very high frame features. As discussed above, videos have different patterns when downsampled to different numbers of frames. A subvideo of one frame has totally different feature patterns than a subvideo of 100 frames, even if the two subvideos are from the same video. This introduces extra learning difficulty. One idea for solving this takes inspiration from previous works and combines it with our work. CKConv [9] and FFN [7] propose networks that adapt to inputs with different shapes, increase their ability to address the discrepancy between different input shapes. By integrating their idea and our training method, we believe that this problem can be solved.

## 7. Conclusions

In current video classification research, models are trained with a conventional paradigm, which means that 3D-CNN models can only function at one specific temporal length. This introduces many inconveniences when applying models in practice. In this work, we introduce the VLT training paradigm for video models, which includes sampling twice, temporal packing, and SV-Ind Conv. All these operations are simple in implementation and efficient during training. Moreover, a consistency loss is introduced to further refine the representation. Our method grants models the ability to operate at a much wider range of temporal lengths while improving their classification accuracy. Unlike previous works that attempted to solve this problem by adjusting the model structure when provided with different frame settings, our method does not alter the models during testing in any way, making it easily generalizable to any 3D-CNN model. The advantages of different training methods are shown in Table 11. Compared with conventional training, our method can function at different temporal lengths. Compared with other flexible training paradigms, our method achieves cheaper training and implementation. Moreover, our work demonstrates that while current deep models have unknown potential, this potential is held back by a flawed training paradigm. By simply altering the training method, the ability of models to handle videos of varying lengths can be unlocked without adding any new parameters. We hope that this work will encourage more researchers to develop better training methods.

## Figures and Tables

**Table 1:** Comparison of accuracy and execution speed on CPU; Exe. Time stands for execution time, and the accuracy of eight-frame and sixteen-frame inputs on Diving48 [1] and Gym99 [2] are reported. All methods used ResNet18 [15] as the backbone.

Model	Exe. Time (8f|16f)	Div48 Acc. (8f|16f)	Gym99 Acc. (8f|16f)
MVF [24]	161 ms|279 ms	73.2|80.2	88.5|91.7
ActionNet [25]	272 ms|455 ms	74.3|83.2	89.0|92.3
TP Conv	141 ms|250 ms	74.0|81.1	88.6|91.6

**Table 2:** Comparison with the conventional training paradigm on Diving48 [1]. Here, (*i*)f-model stands for the model trained with *i* number of frames and with the conventional paradigm, Ensemble denotes the ensemble of all models, where we picked the designated model accordingly at different frame numbers, and the different columns demonstrate the performance at different frame settings during evaluation. ‘# of Frames’ represents ‘number of Frames’.

Model/# of Frames	8	10	12	14	16	δ
8f-model	73.9	75.3	75.6	74.6	73.3	−3.9
10f-model	74.0	77.7	77.7	78.7	78.6	−1.1
12f-model	72.1	76.5	78.7	78.5	78.8	−1.5
14f-model	70.3	75.0	78.5	80.9	80.0	−1.4
16f-model	69.8	74.9	76.9	80.9	81.1	−1.7
Ensemble	73.9	77.7	78.7	80.9	81.1	0
VLT-model	77.1	79.7	81.9	81.5	82.3	+2.0

**Table 3:** Comparison with conventional training paradigm on Gym99 [2]. Here, (*i*)f-model stands for the model trained with *i* number of frames and with the conventional paradigm, Ensemble denotes the ensemble of all models, where we picked the designated model accordingly at different frame numbers, and the different columns demonstrate the performance at different frame settings during evaluation. ‘# of Frames’ represents ‘number of Frames’.

Model/# of Frames	8	10	12	14	16	δ
8f-model	88.6	88.6	87.3	83.9	79.1	−4.8
10f-model	87.8	89.9	90.4	89.1	87.0	−1.5
12f-model	85.2	89.0	90.8	90.3	89.7	−1.3
14f-model	81.8	87.8	90.2	91.0	91.1	−1.9
16f-model	76.9	85.5	89.1	90.7	91.5	−3.6
Ensemble	88.6	89.9	90.8	91.0	91.5	0
VLT-model	89.7	90.9	91.3	91.8	92.0	+0.8

**Table 4:** Comparison with state-of-art methods on Diving48. The upper part records the performance of an ensemble of 8-frame to 16-frame models with conventional training in Table 2. The middle part (VVLT-iter ∼ VLT-$S_{8–16}$) includes the methods dedicated to processing frame ranges between 8 and 16. The lower part (FFN and VLT-$S_{4–16}$) shows the methods dedicated to processing frame ranges between 4 and 16. VLT-$S_{n–m}$ denotes the VLT method trained with a range of frame numbers from *n* to *m*. ‘# of Frames’ represents ‘number of Frames’.

Model/# of Frames	4	8	10	12	14	16	δ
Ensemble	55.3	74.0	77.7	78.8	80.9	81.1	0
VVLT-iter.	-	73.1	77.1	79.8	80.2	79.8	−0.4
VVLT-epoch	-	72.2	76.4	78.3	79.2	78.5	−1.5
MutualNet [8]	-	69.5	73.8	77.1	78.5	79.6	−2.7
VLT-$S_{8–16}$	-	77.1	79.7	81.9	81.5	82.3	+2.0
FFN [7]	58.7	76.1	78.2	79.9	81.1	82.6	+1.4
VLT-$S_{4–16}$	58.0	76.0	78.9	80.9	81.1	82.0	+1.5

**Table 5:** Comparison with state-of-art methods on Gym99. The upper part records the performance of an ensemble of 8-frame to 16-frame models with conventional training in Table 3. The middle part (VVLT-iter ∼ VLT-$S_{8–16}$) includes the methods dedicated to processing frame range between 8 and 16. Thelower part (FFN and VLT-$S_{4–16}$) shows the methods dedicated to processing frame range between 4 and 16. VLT-$S_{n–m}$ denotes the VLT method trained with a range of frame numbers from *n* to *m*.

Model/# of Frames	4	8	10	12	14	16	δ
Ensemble	82.2	88.6	89.9	90.8	91.0	91.5	0
VVLT-iter.	-	86.4	89.1	89.9	89.5	89.4	−1.5
VVLT-epoch	-	87.6	89.6	89.7	89.0	86.9	−1.8
MutualNet [8]	-	79.5	86.9	89.8	90.9	91.4	−2.66
VLT-$S_{8–16}$	-	89.7	90.9	91.3	91.8	92.0	+0.8
FFN [7]	83.0	89.7	89.9	90.5	90.8	91.1	+0.2
VLT-$S_{4–16}$	82.5	89.7	90.3	91.0	91.5	91.7	+0.5

**Table 6:** Comparison of different training methods in terms of training complexity, evaluated with ResNet18 on an Nvidia 2080Ti. The upper part, denoted Con. Train (16f-model), represents a 16-frame model trained with the conventional method, with the flexible training methods shown below. Training FLOPs denotes the number of floating point operations during training, while Num. of Param. stands for number of parameters. GPU Hours refers to the time needed to train the model on a GPU; the GPU memory was recorded by Pytorch with one training sample on each GPU. For GPU Hours, the training time on both Diving48 (left) and Gym99 (right) was recorded.

Method	Train FLOPs	Num. of Param.	GPU Hours	GPU Memory
Con. Train (16f-model)	30.4 G	11.3 M	16.7|23.6	1.7 G
Ensemble	110.4 G	56.5 M	62.5|89.3	1.7 G
MutualNet [8]	49.7 G	11.3 M	17.6|27.4	1.8 G
FFN [7]	51.6 G	11.5 M	27.1|45.5	2.2 G
VLT-$S_{8–16}$	44.2 G	11.3 M	22.9|34.1	1.9 G
VLT-$S_{4–16}$	36.8 G	11.3 M	18.7|28.4	1.8 G

**Table 7:** Comparison of different flexible training methods in terms of implementation complexity; here, Modify model in training/test? indicates whether the model needs modification during training/testing.

Model	Modify Model in Training?	Modify Model in Testing?
Ensemble	✔	✔
MutualNet [8]	×	×
FFN [7]	✔	✔
VLT	✔	×

**Table 8:** Ablation study of consistency loss on Diving48.

Method/# of Frames	8	10	12	14	16	δ
Ensemble	74.0	77.7	78.8	80.9	81.1	0
α=0	74.9 (+0.9)	78.1 (+0.4)	80.5 (+1.7)	80.9 (+0.0)	81.7 (+0.6)	+0.7
α=0.1	75.3 (+1.3)	78.5 (+0.8)	80.5 (+1.7)	81.1 (+0.2)	81.4 (+0.3)	+0.9
α=0.2	75.9 (+1.9)	78.2 (+0.5)	79.3 (+0.5)	81.1 (+0.2)	81.4 (+0.3)	+0.7
α=0.3	77.0 (+3.0)	79.3 (+1.6)	80.1 (+1.3)	80.9 (+0.0)	81.4 (+0.3)	+1.3
α=0.4	77.1 (+3.1)	79.7 (+2.0)	81.9 (+3.1)	81.5 (+0.6)	82.3 (+1.2)	+2.0
α=0.5	76.5 (+2.5)	78.7 (+1.0)	81.0 (+2.2)	80.0 (−0.9)	80.5 (−0.6)	+0.8

**Table 9:** Ablation study of consistency loss on Gym99.

Method/# of Frames	8	10	12	14	16	δ
Ensemble	88.6	89.9	90.8	91.0	91.5	0
α=0	89.1 (+0.5)	90.5 (+0.6)	91.5 (+0.7)	91.4 (+0.4)	91.7 (+0.2)	+0.5
α=0.1	89.5 (+0.9)	90.6 (+0.7)	91.2 (+0.4)	91.3 (+0.3)	91.5 (+0.0)	+0.4
α=0.2	89.5 (+0.9)	90.8 (+0.9)	91.4 (+0.6)	91.9 (+0.9)	92.0 (+0.5)	+0.8
α=0.3	89.4 (+0.8)	90.8 (+0.9)	91.4 (+0.6)	91.4 (+0.4)	91.9 (+0.4)	+0.6
α=0.4	89.7 (+1.1)	90.9 (+1.0)	91.3 (+0.5)	91.8 (+0.8)	92.0 (+0.5)	+0.8
α=0.5	89.2 (+0.6)	90.6 (+0.7)	91.0 (+0.2)	91.4 (+0.4)	92.0 (+0.5)	+0.5

**Table 10:** Results on Something-Something V1; CT stands for the conventional training method.

Model	Training Method	# of Frames	Top1
TSM [3]	CT	8	45.6
TSM [3]	CT	16	48.6
TSM [3]	VLT	8	47.9
TSM [3]	VLT	16	50.6
MVF [24]	CT	8	48.8
MVF [24]	CT	16	50.0
MVF [24]	VLT	8	50.3
MVF [24]	VLT	16	51.6

**Table 11:** Conclusions showing the advantages of different training methods. CT stands for conventional training, while En. CT stands for the ensemble of conventional training models.

	CT	En. CT	MutualNet [8]	FFN [7]	VLT
Function at varying temporal length	×	✔	×	✔	✔
Low training cost	✔	×	✔	×	✔
Simple implementation	✔	×	✔	×	✔

## Data Availability

Data are available in a publicly accessible repository. The original data presented in the study are openly available at [1,2,28,29,30,32].
